# The influence of a home-based exercise intervention on human health indices in individuals with chronic spinal cord injury (HOMEX-SCI): study protocol for a randomised controlled trial

**DOI:** 10.1186/s13063-016-1396-z

**Published:** 2016-06-08

**Authors:** Tom E. Nightingale, Jean-Philippe Walhin, James E. Turner, Dylan Thompson, James L. J. Bilzon

**Affiliations:** Department for Health, University of Bath, BA2 7AY Bath, UK

**Keywords:** Aerobic capacity, Metabolic health, Paraplegia, Spinal cord injury, Physical activity, Immune function, Inflammation, Gene expression, Exercise intervention

## Abstract

**Background:**

Spinal cord injury (SCI) creates a complex pathology that can lead to an increase in sedentary behaviours and deleterious changes in body composition. Consequently, individuals with SCI are at increased risk of developing cardiovascular disease and type-2 diabetes mellitus. While the role of physical activity on the reduction of chronic disease risk is well documented in non-disabled individuals the evidence is less conclusive for persons with SCI. The aim of this methodological paper is to outline the design of a study that will assess the role of a home-based exercise intervention on biomarkers of metabolic and cardiovascular health in persons with SCI: the HOMEX-SCI study.

**Methods/design:**

Eligible participants will be inactive (physical activity level ≤1.60) individuals, with a chronic (more than 1 year) spinal cord lesion between the second thoracic and the fifth lumbar vertebrae, and aged between 18 and 65 years. Following baseline laboratory testing and lifestyle monitoring, participants will be randomly allocated to a control (CON) group or a 6-week home-based exercise intervention (INT) group. The INT consists of 45 minutes of moderate-intensity (60–65 % peak oxygen uptake) arm-crank exercise four times per week. Participants assigned to the CON group will be asked to maintain their normal lifestyle. The main outcomes of this study (biomarkers of metabolic and cardiovascular health) are obtained from venous blood samples, collected in the fasted and postprandial state. Eight other measurement categories will be assessed: (1) body composition, (2) physical activity, (3) energy intake, (4) measures of health and wellbeing, (5) resting metabolic rate, heart rate and blood pressure, (6) aerobic capacity, (7) immune function, and (8) adipose tissue gene expression.

**Discussion:**

This study will explore the feasibility of home-based moderate-intensity exercise and ascertain its impact on metabolic and cardiovascular health in comparison to a lifestyle maintenance CON group. Findings from this study may help to inform new evidence-based physical activity guidelines and also help to elucidate the physiological mechanisms whereby exercise might exert beneficial effects in persons with chronic SCI. The results will also act as a scientific platform for further intervention studies in other diverse and at-risk populations.

**Trial registration:**

International Standard Randomised Controlled Trial Number: ISRCTN57096451. Registered on 11 July 2014.

## Background

A spinal cord injury (SCI) is a significant life-changing event which has wide-ranging implications for multiple physiological systems. There are no reliable estimates of the global prevalence of SCI, perhaps reflecting the need for improvements in international medical standards and guidelines for reporting SCI. Over the past 60 years there has been a worldwide improvement in the acute survival of patients with traumatic SCI through the possibility of rapid transportation to a specialised unit, medical treatment advancements and improved rehabilitation [1]. As a consequence there has been a shift in focus from acute life support medicine, to addressing other secondary health complications and comorbidities associated with ageing with paralysis [2, 3]. Consequently the long-term demands on medical and support resources are high. A recent systematic review examining survival worldwide after SCI concluded that overall mortality in SCI is up to three times higher than in the general population [4]. Evidence now suggests that cardiovascular disease (CVD) is the leading cause of mortality in individuals with chronic SCI [5]. Besides CVD, epidemiological studies have also revealed the incidence of type-2 diabetes mellitus (T2DM) to be high in individuals with SCI [6–8]. Indeed, it has been suggested that adults with SCI are four times more likely to develop T2DM than non-disabled controls [9].

The contribution of regular physical activity (PA) to reduce the risk of these chronic diseases is well documented and broadly accepted in the non-disabled population [10, 11]. In individuals with SCI, involvement in sports and recreation is often restricted by the loss of voluntary motor control, as well as autonomic dysfunction and early onset of skeletal muscle fatigue [12–14]. There are also numerous psychosocial and environmental barriers: reduced self-esteem, lack of accessible facilities, unaffordable equipment, fear of injury and/or excessive parental or care protection, which have all been cited as barriers to engagement in PA [15–18]. It is likely that the adoption of a more sedentary lifestyle [19–21], in conjunction with reduced physical function [22], and adverse changes in body composition [23, 24] (e.g. lower limb skeletal muscle atrophy [25, 26]) after SCI, contribute to the increased morbidity and mortality associated with this population.

There is convincing evidence to suggest that component risks for the development of metabolic syndrome occur at a heightened frequency in individuals with SCI; specifically, increased central obesity [27–29], impaired fasting glucose/T2DM [30, 31] and dyslipidaemia [32]. The accumulation of excess adiposity is thought to be associated with a sustained positive energy balance secondary to reductions in resting metabolic rate and physical activity energy expenditure. This is associated with local and systemic inflammation, with studies demonstrating a two to threefold increase in the levels of circulating inflammatory markers in persons with SCI compared to non-disabled persons [33–35]. Although elevated chronic inflammation is not formally included among evidence-based metabolic syndrome components, its role in the development of atherosclerosis has been extensively characterised [36–38]. Although it was previously thought that adipose tissue was simply a store for energy, this dynamic tissue is now recognised as an endocrine organ [39–41]. Adipose tissue secretes a number of hormones, collectively termed ‘adipokines’, which play a key role in regulating glucose metabolism and insulin sensitivity, as well as governing aspects of immune function and a variety of other physiological processes. Indeed, the expression of many adipokines is markedly dysregulated with excess adiposity [42] and in individuals with SCI [43, 44]. Although a causal link has not been robustly confirmed, several aspects of immune function are also impaired in sedentary, overweight and obese individuals [45–49] and it is possible that these findings may generalise to individuals with SCI [50].

### The impact of physical activity on health in persons with spinal cord injury

Various biological mechanisms, integral in the maintenance of metabolic control, are influenced by physical inactivity and have been implicated in the progression of certain chronic diseases [51]. To date, cross-sectional evidence has been used to inform PA guidelines for individuals with SCI. These disability-specific guidelines [52], developed as part of the Study of Health and Activity in People with Spinal Cord Injury (SHAPE SCI) [53, 54], propose that at least 20 minutes of moderate- to vigorous-intensity aerobic activity should be undertaken twice a week by people with SCI. These guidelines were developed through a systematic and critical appraisal of research to date, which was then deliberated by a multidisciplinary expert panel to assess the quality of the evidence. The comprehensive systematic review to compile this evidence indicated that most studies informing these guidelines were of poor quality and focused solely on physical capacity and muscular strength [54]. Therefore, there is a lack of empirical evidence regarding the most appropriate dose of exercise or physical activity parameters (e.g. frequency, duration) for improving metabolic health in this population.

The current literature on PA research for individuals with SCI has recently been systematically classified [55]. Most studies between 2000 and 2012 have been categorised as either: phase 1 (linking PA and health outcomes); phase 2 (validating or developing PA monitors) or; phase 3 (identifying factors influencing behaviour or examining theories of behaviour change). Such categorisation implies that this field is in the early stages of development and research should now focus on phase 4 (evaluating interventions) and phase 5 (disseminating health promotion policies and translating research into practice). Due to the inconsistent findings across studies, concluded via a systematic review requested by the Consortium for Spinal Cord Medicine [56], it would appear that current evidence is insufficient to determine whether exercise improves carbohydrate and lipid metabolism disorders among adults with SCI. However, previous studies have demonstrated that arm-crank exercise improves lipid profiles [57], inflammatory biomarkers [58] and fasting insulin concentrations [59] with interventions ranging from 12 to 16 weeks in duration. It is possible that inconsistencies in the current literature may be caused by considerable heterogeneity in study participants, the type of exercise programme and/or the outcome measures examined. Here we describe the study design for a randomised controlled trial (RCT) with a homogeneous cohort, examining a variety of outcome measures not simply confined to physical capacity, that will contribute towards our understanding of how a home-based upper body exercise intervention might impact metabolic, cardiovascular and immunological health in individuals with SCI: the HOMEX-SCI study.

### Objectives

The primary objective of the HOMEX-SCI study is to assess the impact of a 6-week home-based moderate-intensity arm-crank exercise intervention on markers of metabolic and cardiovascular health in inactive individuals with chronic SCI. Little is known about the impact of a moderate- to vigorous-intensity exercise intervention on messenger ribonucleic acid (mRNA) expression of adipose tissue, which is surprising considering that it is a major site for energy storage. To our knowledge, adipose tissue biology has never been studied in a cohort of individuals with SCI. Thus, a secondary objective is to investigate at baseline, and following intervention, the expression of genes within adipose tissue that are associated with a variety of biological processes such as energy homeostasis, glucose metabolism, lipid metabolism and inflammatory responses. Other objectives include: characterising aspects of adaptive immune function pre and post intervention, quantifying changes in body composition, aerobic capacity, dietary and physical activity behaviours and various constructs of health and wellbeing. The exercise intervention group’s responses, for all outcome measures over 6 weeks, will be compared to a control group.

## Methods

### Study design

**Fig. 1:**
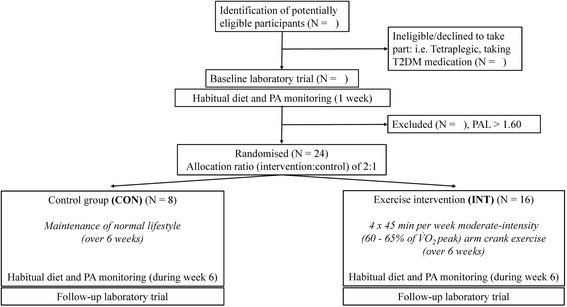
Study flow diagram (Abbreviations: *CON* control group, *INT* exercise intervention group, *PA* physical activity, *PAL* physical activity level, *T2DM* type-2 diabetes mellitus, *V̇O*_*2*_ *peak* peak oxygen uptake)

This study is a pre-post, parallel-group design, with participants randomly assigned to a 6-week home-based exercise intervention (INT) or a control period (CON). The planned experimental design is summarised below (Fig. 1), consistent with current Consolidated Standards of Reporting Trials (CONSORT) guidelines [60]. The South West (Exeter) National Research Ethics Service Committee has approved this study protocol (REC reference number 14/SW/0106) and the trial has been registered as a current controlled trial (ISRCTN57096451). Upon initial contact (by email or telephone), the study will be described briefly and those interested in enrolling will be asked to read a detailed participant information sheet and complete a health screen questionnaire. Interested individuals will be screened via a telephone conversation 48 hours after their initial contact to ascertain eligibility. A focus will be placed on establishing a good rapport at this initial contact and also as individuals progress through the enrolment process. Written consent will be obtained during the initial laboratory visit and participants will be informed that they can withdraw from the study at any time without consequence.

Baseline laboratory testing will be performed in the Centre for DisAbility Sport and Health (DASH) at the University of Bath approximately 2 weeks before the INT/CON period commences. During this 2-week interim period participants will be instructed not to alter their normal activity patterns or dietary behaviours. This period permits a 7-day estimation of free-living habitual energy balance, whereby PA is monitored and participants are asked to maintain a detailed weighed record of food intake. The additional time accounts for participants returning the activity monitors, and downloading and subsequent analysis of data to ascertain participant eligibility. A member of the research team will also travel to each participant’s home to deliver a portable arm-crank ergometer and supervise the first exercise session. Importantly, the interim period also allows exactly 8 weeks between baseline and follow-up testing, which ensures that eumenorrheic female participants will be within the follicular phase of their menstrual cycle (3–10 days after the onset of menses) during both laboratory visits. Controlling for menstrual cycle stage is important as hormone fluctuations can influence insulin sensitivity [61].

### Recruitment and randomisation

It has been reported that recruitment for a RCT, especially in the absence of direct access to a clinical population, requires considerably more resources and time than initially anticipated in order to achieve adequate enrolment [62]. In a systematic review, Ross et al., [63] described patient barriers to recruitment for RCTs. These included: (1) additional demands of the trial increasing participant burden and (2) patient preferences for or against a particular treatment. Barriers to research participation are perhaps even more exaggerated for people with disabilities due to complex health problems [64], lack of transportation [65], cognitive impairments and financial stress [66]. With this in mind, we plan to deliver the intervention by setting up arm-crank ergometers in participants’ own homes, thereby minimising any potential accessibility/transport barriers to participants becoming more active. Transportation needs are a major participation barrier for individuals with disabilities, and so travel expenses to the University of Bath for laboratory testing will be reimbursed. Recruitment material will be promoted by nationwide charities and circulated on Internet forums and social networking sites. Previous Centre for DisAbility Sport and Health research participants who met the inclusion criteria of the trial have been notified directly about the study via email.

After baseline laboratory testing and habitual free-living monitoring, the first nine participants will be randomly allocated to the experimental group (INT) or the CON group using a block randomisation plan (fixed block size of nine; allocation ratio of 2:1; no stratification). Randomisation will be performed by an independent researcher using a concealed list generated with a web-based platform (www.randomization.com). Within a highly heterogeneous population such as individuals with SCI, we decided to use minimisation [67] to ensure balance between the two groups for baseline characteristics (age, body mass, level of spinal cord lesion and physical activity level). Subsequent participants who enrol on the trial will, therefore, be allocated to either the INT group or the CON group in order to balance the groups in these variables of interest. We opted for an unequal allocation ratio as it has been supported as an alternative approach for ‘confirmatory trials’ [68, 69], primarily to increase the amount of data on the INT, where individual responses are likely more variable. We presume limited variability in the CON group, as participants will continue to live their normal lifestyle. Any small loss of precision with the use of unequal allocation has been appropriately accounted for in the sample size calculation. While participants may have a strong preference to receive the INT and not be in the CON group, we will try to overcome this by offering a waiting-list control. Participants in the CON group will, therefore, be offered the opportunity to participate in the INT once they have completed the CON period.

### Participants and eligibility criteria

The participants will be inactive individuals (physical activity level; PAL ≤1.60), with a chronic (more than 1 year) spinal cord lesion between the second thoracic and the fifth lumbar vertebrae, who are aged between 18 and 65 years. Volunteers with neurologically incomplete injuries will be considered eligible if they are wheelchair users for more than 75 % of their waking day. Participants will only be included if they have been weight stable (±3 kg) for at least 6 months and have no plans to change their diet or exercise behaviours. Individuals self-reporting active medical issues including pressure sores, urinary tract infections, cardiac disorders, cardiovascular contra-indications for testing [70] or musculoskeletal complaints of the upper extremities will be excluded. Participants on T2DM medication will also be excluded.

Habitual PAL will be estimated over a representative 7-day period following the baseline laboratory visit using a wearable PA-monitoring device (Actiheart™, Cambridge Neurotechnology Ltd., Papworth, UK), which integrates accelerometer and heart rate signals. This device has previously been validated for use in this population [71]. Participants will wear this monitor continuously (24 hours a day) and will be instructed only to remove it when showering or bathing. Individuals with a PAL >1.60 will be precluded from progressing to the group allocation stage.

### Main trial day protocol

**Fig. 2:**
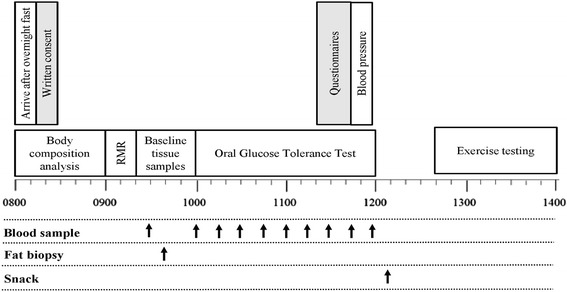
Schematic overview of main trial day timeline, with 08.00 arrival time of the participant as an example (Abbreviations: *RMR* resting metabolic rate)

The same experimental procedures will be completed on both baseline and follow-up trial days. Participants will arrive at the Centre for DisAbility Sport and Health laboratory at 08.30 ± 1 h, following an overnight fast (at least 10 h). Twenty-four hours prior to each main laboratory trial day, participants will abstain from strenuous exercise, caffeine (tea/coffee) and alcohol. The final exercise bout prescribed in the INT group will be more than 36 h prior to follow-up testing in order to reduce the acute effects from the previous bout of exercise. Participants will also be asked to consume 1 pint of water on the morning of testing to ensure adequate hydration. See Fig. 2 for the schematic overview of the main trial day timeline.

#### Body composition analysis

After being asked to void, body mass will be measured, with participants wearing light clothing, using platform wheelchair scales (Detecto® BRW1000, Webb City, MO, USA). The wheelchair, along with participants’ shoes will be weighed separately and subtracted from the total mass of the participant to derive an accurate body mass as recommended [72]. For body composition analysis, participants will transfer onto a Dual-energy X-ray Absorptiometry (DEXA) scanning table (Discovery, Hologic, Bedford, UK). Supine height will be measured in centimetres along the left hand side of the body using a non-elastic tape measure (Lufkin, Sparks, MD, US). Participants will be positioned centrally on the scanning table with their feet spaced evenly either side of the mid-point of the body, with their arms placed mid-prone with an equal gap to the trunk on both sides. For participants who experience leg spasms, their knees will be flexed at a 45°-angle and supported by a pillow. Participants will be instructed to remain as still as possible. All scans will be conducted following a daily QC scan of a Spine Phantom as per the manufacturer’s instructions.

#### Resting metabolic rate measurement

Resting metabolic rate (RMR) will be estimated by indirect calorimetry from expired air samples collected into 200-L Douglas Bags (Hans Rudolph, Kansas City, MO, USA) via falconia tubing (Baxter, Woodhouse and Taylor Ltd., Macclesfield, UK) with concurrent measurement of inspired air composition; a reliance of standard atmospheric concentrations has recently been discouraged [73]. The process of indirect calorimetry has previously been described in detail [74]. Expired concentrations of oxygen (O_2_) and carbon dioxide (CO_2_) will be measured, from a known volume of each sample using paramagnetic and infrared analysers, respectively (MiniMP 5200, Servomex Ltd., Sussex, UK). Four 5-min Douglas Bag samples will be collected for RMR in accordance with best practice guidelines [75]. Measurements will be conducted following a 20-min rest and ambient laboratory temperature will be maintained between 20 and 25 °C. RMR will be taken as the average of two or more Douglas Bag samples that are within 100 kcal day^−1^ of each other. Participants will also wear a Polar T31 heart rate monitor (Polar Electro Inc., Lake Success, NY, USA) during RMR measurement; resting heart rate values will be averaged over the 20-min collection period.

#### Blood sampling

After a 15-min supine rest, a cannula (BD Venflon Pro, BD, Helsingborg, Sweden) will be inserted into an antecubital vein, and a 45-ml fasting sample drawn. From this sample, 25 ml of whole blood will be collected into sodium heparin tubes and peripheral blood mononuclear cells (PBMCs) will be isolated using density gradient centrifugation using standard methods. Cells in freezing medium (70 % Roswell Park Memorial Institute (RPMI) media, 20 % fetal bovine serum (FBS), 10 % dimethylsulphoxide (DMSO)) will be frozen at −1 °C/min using a Nalgene ‘Mr Frosty’ container. Samples will be stored at −80 °C and assayed for immunological measurements within 12 months.

The remaining blood from the baseline sample (and subsequent blood samples during the oral glucose tolerance test; see oral glucose tolerance test section below) will divided into separate tubes for obtaining serum and plasma. For serum, whole blood will be placed into a serum separation tube and left to stand at room temperature for 15 min before centrifugation. For plasma, whole blood will be placed into tubes coated with ethylenediaminetetraacetic acid (EDTA) and centrifuged immediately. Samples will be centrifuged (Heraeus Biofuge Primo R, Kendro Laboratory Products Plc., Tyne and Wear, UK) at 5000 rpm for 10 min at 4 °C, with serum/plasma subsequently dispensed into 0.5-ml aliquots and immediately cooled on dry ice and then stored at −80 °C. A small aliquot of EDTA blood will be used to obtain the full leucocyte differential and other haematological variables (SF-300, Sysmex Ltd., Milton Keynes, UK).

#### Adipose tissue biopsy

An adipose tissue sample will be collected from the area around the waist, approximately 5 cm lateral to the umbilicus, using a well-established ‘needle aspiration’ technique [76]. The area will be thoroughly disinfected with Videne, before injection of anaesthetic (approximately 5 ml lignocaine hydrochloride 1 %) into a small area. Five minutes later, a needle will be inserted in the subcutaneous fat tissue around the waist in order to collect approximately 1–2 g of fat tissue. Swabs and pressure will be applied to the area once the sample has been collected. A dressing will then be applied to the area. The sample will be cleaned with isotonic saline and any clots will be manually removed. After the sample has been weighed, it will be homogenized in 5 ml of Trizol (Invitrogen, Paisley, UK) and stored at −80 °C.

#### Oral glucose tolerance test (OGTT)

After the baseline blood sample and adipose tissue biopsy, the participant will consume 113 ml of Polycal (Polycal, Nutricia Advanced Medical Nutrition, Trowbridge, UK) and 87 ml of water, equivalent to 75 g of anhydrous glucose, within 5 minutes. Further 5-ml blood samples will be drawn at 15-min intervals for the next 2 hours. The intravenous cannula is to be kept patent through periodic flushing with 0.9 % NaCl (B. Braun, Sheffield, UK) infusion, with the first 5 ml of each blood draw being discarded. Blood pressure will also be measured during the final 15 min of the OGTT using an automated blood pressure monitor (Boso Medicus Prestige, Bosch + Sohn GmbH, Jungingen, Germany). The short form-36 health survey (SF-36), the EuroQol-5 dimensions, 5 levels questionnaire (EQ-5D-5 L), the Wheelchair User’s Shoulder Pain Index (WUSPI), the Fatigue Severity Scale (FSS) and the Exercise Self-efficacy Scale (ESES) will be administered during the last 30 min of the OGTT.

#### Exercise testing pre and post intervention

Participants will undertake an incremental submaximal arm-crank ergometry test using a portable desktop ergometer (Monark compact rehab 871E, Dalarna, Sweden). This test will comprise three 3-min stages, separated by a 1-min rest period during which the resistance will be increased. Participants will be instructed to maintain a cadence of 75 rpm throughout. A variety of power outputs will be chosen that are sufficient to cover the relative exercise intensities (60–65 % of V̇O_2_ peak) employed during training sessions in the INT group. During the final minute of each stage, V̇O_2_ and heart rate will be recorded to determine the relationships between V̇O_2,_ exercise intensity (work rate) and heart rate. This test will be performed on the same ergometer provided to each participant during the INT.

Peak oxygen uptake (V̇O_2_ peak) will be determined at the end of each laboratory visit using a continuous, progressive intensity test on an electrically braked arm-crank ergometer (Lode Angio, Groningen, Netherlands). A cadence of 75 rpm will be encouraged throughout and a starting intensity will be selected based on the participant’s training history. The resistance will be increased by 14 W every 3 minutes until the point of volitional exhaustion (approximately 9–12 min). Continuous gas exchange measurements will be collected using a TrueOne® 2400 computerized metabolic system (ParvoMedics, Salt Lake City, UT, USA), calibrated according to manufacturer’s instructions prior to use.

### Home-based exercise intervention

The exercise intervention will consist of moderate-intensity (60–65 % V̇O_2_ peak) home-based exercise four times per week on a portable desktop ergometer. The intervention will progress by extending the duration of each session by 5 minutes in the first week, from 30 to 45 min. The intensity will also be increased from approximately 60 % V̇O_2_ peak for the first 3 weeks to approximately 65 % V̇O_2_ peak for the final 3-week period. Participants will be instructed to work at a cadence of 75 rpm and will be able to schedule exercise sessions around their own lifestyle (i.e. no restrictions with regards to training on consecutive or non-consecutive days, weekdays or weekends). The first training session at each participant’s home will be supervised by a member of the research team to ensure that the arm-crank ergometer is set up appropriately and that the correct duration and intensity of exercise is adhered to. Subsequent home-based exercise sessions will be performed under the supervision of a carer or spouse as agreed on the informed consent document. To attain the desired intensity in each session, participants will wear a Polar T31 heart rate monitor (Polar Electro Inc., Lake Success, NY, USA) and will be instructed to adjust the resistance in order to achieve the target heart rate. The target heart rate for each participant will be calculated during baseline testing using V̇O_2,_ work rate and heart rate data collected during the incremental submaximal arm ergometer test. If the V̇O_2_ and heart rate relationship is influenced by a blunted cardiovascular response to exercise in participants with higher-level injuries (above T6) then a target work rate (W), derived from the individual V̇O_2_ and work rate relationship, will be prescribed instead. The intervention will be conducted on the same portable desktop ergometer that submaximal exercise testing was performed on.

Each exercise session will be monitored via a GENEActiv device (GENEActiv, Activinsights, Cambridge, UK) worn on the wrist [77] to determine compliance. Compliance records will be cross-checked with an activity diary where participants record the date, time, duration, difficulty and total revolutions of each exercise session along with average heart rate during the last 10 minutes of exercise. Sampling at 30 Hz, the GENEActiv is capable of recording PA for up to 3 weeks. At the midway point a newly initialised and charged GENEActiv device will be sent to the participant by standard mail. During the final week of the intervention exercise intensity compliance will be checked by heart rate recordings on the Actiheart™ monitor. Adherence to the intervention will be maintained with regular weekly telephone calls and emails. No dietary constraints will be imposed: participants in both groups will be free to consume food and fluid ad libitum throughout.

### Outcome measures

#### Body composition, resting metabolic rate and aerobic capacity

To determine body composition, DEXA scans will be analysed for total and regional (trunk, legs, arms) fat mass, fat free mass, bone mineral content and fat percentage, following the guidelines described in the user manual (Hologic, Bedford, UK). Fasting respiratory exchange ratio (RER) will also be calculated during the resting metabolic rate (kcal day^−1^) measurement. Aerobic capacity will be measured at the point of volitional exhaustion during the incremental arm-crank test. A number of criteria (participants must meet two of these) will be applied to determine whether this endpoint is reflective of a valid V̇O_2_ peak value. These were: (1) a peak RER value ≥1.1, (2) a peak heart rate of 95 % or more of the age-predicted maximum (200 b min^−1^ minus chronological age) and an increase in V̇O_2_ ≤ 2 ml kg^−1^ min^−1^ in response to an increase workload [70]. Moreover, peak workload (W) will be measured at volitional exhaustion.

#### Blood measurements

The metabolic and cardiovascular health, along with immune function variables measured are shown in Table 1. Besides the haematological profile which is measured immediately during the main trial day, these variables will be quantified during a batch analysis once the final participant has completed the study. In order to simplify data analysis and facilitate the interpretation of a complex data set [78, 79], serial measurements of glucose and insulin responses to the OGTT at baseline and follow-up will be converted into simple summary statistics [80], such as incremental area under the curve (iAUC) [81] and insulin sensitivity index (ISI_Matsuda_) [82]. The Homeostasis Model Assessment (HOMA) calculator, incorporating the updated HOMA-2 model [83], will be used to derive fasting estimates of pancreatic β-cell function, insulin resistance and sensitivity.

**Table 1:** Metabolic health, cardiovascular disease risk and immune function variables

Haematological profile	Analytical techniques
Red blood cell, white blood cell, neutrophil, lymphocyte, monocyte and platelet counts, haemoglobin concentrations, haematocrit and haematic indices	Automated haematology system (SF-300, Sysmex Ltd., Milton Keynes, UK)
Biochemical variables	Analytical techniques
Triglycerides, total cholesterol, NEFA, HDL-C, LDL-C, glucose	Automated analyser (Randox RX Daytona, Randox Laboratories, Co. Antrim, UK), in accordance with manufacturer’s instructions using commercially available immunoassays (Randox Laboratories, Co. Antrim, UK).
Insulin	ELISA (Mercodia AB, Uppsala, Sweden)
Adipokines	Analytical techniques
Leptin and adiponectin	ELISA (Quantikine HS, R&D Systems Inc., Abingdon, UK)
Inflammation	Analytical techniques
IL-6	ELISA (Quantikine HS, R&D Systems Inc., Abingdon, UK)
CRP	Automated analyser (Randox RX Daytona, Randox Laboratories, Co. Antrim, UK),
Immune function	Analytical techniques
T cell anti-viral IFN-γ production	Human IFN-γ ELISpot^BASIC^ (Mabtech, Nacka Strand, Sweden)

*CRP* C-reactive protein, *ELISA* enzyme-linked immunosorbent assay, *ELISpot* enzyme-linked immunospot, *HDL-C* high-density lipoprotein cholesterol, *IFN-γ* interferon gamma, *IL-6* interleukin-6, *LDL-C* low-density lipoprotein cholesterol, *NEFA* non-esterified fatty acid

#### Adipose tissue gene expression

The aqueous phase of stored adipose tissue samples will be isolated and total ribonucleic acid (RNA) extracted using the standard protocol supplied with RNeasy mini columns (Qiagen, Crawley, UK). Approximately 2 μg of total RNA will be reverse transcribed using a high-capacity complementary deoxyribonucleic acid (cDNA) reverse transcript kit (Applied Biosystem, Warrington, UK). Pre-designed primers and probes will be obtained from Applied Biosystems for each gene tested. Real-time polymerase chain reaction (PCR) will be performed using a StepOne™ (Applied Biosystems, Warrington, UK).

#### Measures of health and wellbeing

Quality of life and health status will be measured using the SF-36 [84] and EQ-5D-5 L [85] self-report questionnaires, with certain questions adapted for wheelchair propulsion instead of ambulation. Compared with four other instruments used to measure health-related quality of life in persons with SCI, Leduc and Lepage [86] reported better validity of the SF-36 with regard to quantifying the health status of participants. Original responses will be transformed into recorded values (taking into account items which were negatively scored) and specific questions will be averaged to return a value for general health using the RAND 36-Item Health Survey 1.0 scoring method. This value will range from 0 to 100, with 100 representing the best health possible. The EQ-5D-5 L was developed by the EuroQol group and is applicable to a wide range of health conditions, including individuals with SCI [87].

Shoulder pain will be assessed using the WUSPI [88]. The performance-corrected WUSPI score (PC-WUSPI) will be used as it accommodates participants who are unable to undertake certain functions (i.e. item 13: driving?). This correction multiplies the average response for all items by the number of questions attempted, with higher values indicating a greater degree of perceived shoulder pain. The FSS will be administered to measure the severity of fatigue and its effects on certain behaviours. The FSS was initially developed to be used in multiple sclerosis patients [89], but has also been shown to have acceptable test-retest reliability and validity in 48 community living individuals with SCI [90]. Self-efficacy will be assessed using the SCI ESES [91], which was developed specifically to cover issues associated with this unique population.

### Physical activity energy expenditure

Daily physical activity energy expenditure will be estimated before and during the final week of the INT/CON period using an Actiheart™ monitor. Entering heart rate and corresponding J min^−1^ kg^−1^ (derived from indirect calorimetry) data measured throughout the trial day (at rest and during a range of submaximal/maximal exercise intensities) into the manufacturer’s software permits the individual calibration of this device. This method has been previously validated for use in wheelchair users [71]. Raw acceleration signals (expressed as g min^−1^) will also be recorded at baseline and follow-up using a GENEActiv device worn on the wrist [77]. The number of days required to assess habitual PA using wearable devices has been discussed at length [92]. The consensus was that monitoring for 3–4 days is required to achieve 80 % reliability in activity counts. In our work we decided to use an inclusion criteria of 4 days or more (providing one of these days was a weekend day). This criteria also ensures that we capture more PA behavioural information than the physical activity recall assessment for people with a spinal cord injury (PARA-SCI: 3-day recall telephone interview) which has been widely used to quantify free-living PA in wheelchair users [53, 93]. A valid day will require at least 80 % of data for that 24-h period. Data will also be expressed as minutes spent in different intensities of activities on the basis of metabolic equivalents (METs): sedentary <1.5 METs, light- 1.5–2.9 METs, moderate- 3.0–6.0 METs, and vigorous-intensity activity >6.0 METs.

### Energy intake

Participants will be asked to keep a detailed record of their food and fluid intake for a ‘typical’ 7 days. Previous work has demonstrated variation between weekday and weekend energy intake [94]; therefore, 7 days is a suitable period to allow accurate representation of habitual energy intake. Each participant will receive a set of weighing scales (PL11B Digital Scale, Smart Weigh, Chestnut Ridge, NY, USA) to accurately weigh and record foodstuffs, which negates any potential errors in the estimation of food weight [95]. Martin et al., [96] have shown that weighed food records are a more valid measure of energy intake than dietary recall methods. Diet records will subsequently be analysed using Nutritics software (Nutritics Ltd., Dublin, Ireland), to estimate energy intake and macro-nutrient composition.

### Statistical analysis

Changes in key outcome variables within and between trials will be analysed with two-way (group [INT, CON] × day [baseline, follow-up]) and three-way (group [INT, CON] × day [baseline, follow-up × time]) mixed-model analysis of variance (ANOVA). Where significant interactions are observed, multiple *t* tests will determine the location of variance both between treatments at each time point and between time points within each treatment relative to baseline. Both methods will be subjected to a Holm-Bonferroni stepwise adjustment [97]. Standardised effect sizes (Cohen’s *d*) will also be calculated for all variables. Based upon the magnitude of correlation between trials, thresholds of >0.2 (small), >0.5 (moderate) and >0.8 (large) have been suggested [98]. This provides an interpretation of the size of the effects in our outcome measures when using a parallel-group’s study design. Magnitude-based inferences will also be calculated to examine the impact of exercise on all variables of interest.

#### Power calculation and sample size

The sample size was calculated using statistical software (G*Power 3.1.5) on the main outcome measure, fasting serum insulin concentration. This calculation was based on data from a hand-cycling training group, which reported an improvement of −14.3 ± 12.7 pmol l^−1^ [59] in fasting insulin over 16 weeks. Based upon these data (Cohen’s *d* = −1.1) it was estimated that nine participants are required to detect a statistically significant change in insulin sensitivity in the INT group. The power was 0.8 and the alpha was set at 0.05. As a 2:1 ratio is to be adopted, a computer programme (www.statstodo.com/SSizUnequal_Pgm.php) was used to calculate sample size adjustments for two groups with unequal size, to account for any consequences of unequal allocation on statistical power [99]. Also, taking into account an expected drop-out rate of approximately 15 %, we aim to recruit at least 24 (INT: 16, CON: 8) participants with chronic paraplegia.

## Discussion

To our knowledge, this will be the first study to simultaneously evaluate the efficacy of a home-based arm-crank ergometry exercise intervention and explore the biological mechanisms of action in persons with chronic SCI. Considering the aforementioned barriers to engage in PA, a convenient and accessible form of exercise is necessary to maximise exercise compliance in this population. A 16-week laboratory-based RCT [59], which experienced large drop-out rates, has recently advocated that researchers should consider how to make exercise interventions more feasible to individuals with SCI. Home-based arm-crank ergometer exercise has been examined in other hard-to-reach groups (polio patients) [100] and will overcome transportation barriers and the lack of accessible exercise equipment. Changes in outcome measures with increased PA, achieved through enhanced compliance, may indicate that home-based arm-crank exercise has the potential to be used as a more long-term behavioural strategy to improve clinical outcomes and quality of life in persons with SCI.

Various exercise intervention protocols have been employed for individuals with SCI previously, including arm-crank ergometry [101], functional electrical stimulation (FES) [102] or wheelchair propulsion [103] ranging from between 5 to 57 weeks in duration. The frequency of exercise in these studies was typically between two and three sessions per week lasting 30 to 60 minutes per session. We settled on the proposed intervention of 6 weeks of arm-crank exercise, 4 × 45 minutes per week, in order to provide a considerable exercise stimulus. Over this time-course we expect to see improvements in our primary outcome measure of metabolic control, irrespective of weight loss (which may be negligible as a result of a compensatory increase in energy intake) [104].

Previous work has demonstrated that genes expressed in skeletal muscle, related to both ‘insulin action’ and ‘adipocytokine signalling’ pathways, are downregulated after 3 weeks of deconditioning in able-bodied males and upregulated after 6 weeks of FES exercise training in individuals with paralysis [105]. Building on these findings, we speculate that 6 weeks of arm-crank exercise will elicit favourable alterations in the expression of key genes involved in energy homeostasis, glucose metabolism, lipid metabolism and inflammatory pathways in adipose tissue. By examining the expression of genes involved in a variety of biological pathways, we hope to further our understanding of the mechanisms whereby PA might influence metabolic control and inflammation in this population.

It is well established that high levels of habitual PA and/or undertaking structured bouts of exercise can improve aspects of immune function in healthy individuals without a disability [48, 49, 106, 107]. However, it is unknown whether these findings generalise to people with SCI. Considering that: individuals with SCI exhibit less robust immunity compared to regularly active, lean and able-bodied people [50]; and that impaired immune function has been reported in sedentary overweight individuals without a disability [45–47], then it is possible that disability-associated impairments in immune function are brought about by sedentary behaviour and adipose tissue accumulation/deregulation. This idea has never been investigated. In the proposed work, we will examine the magnitude of anti-viral cell-mediated immunity (i.e. T cell IFN-γ production) in resting samples pre and post intervention. This response is a functionally relevant and readily observed measure of host immunity that can be assessed sensitively with robust methodology.

The findings from this study might help to inform new evidence-based PA guidelines specific to individuals with a chronic SCI. By taking a holistic approach that addresses a number of relevant outcome measures in a single study, using a rigorous research design (RCT with true control group), this study is in accordance with recent recommendations [56, 108]. The results will also act as a scientific platform for further interventional studies in other diverse and at-risk populations.

### Trial status

Enrolment into the study started on 1 September 2014. As of 2 February 2016, 21/24 participants were recruited. Recruitment is expected to be completed by 29 April 2016 and follow-up assessments in a further 8 weeks.

## Abbreviations

ANOVA, analysis of variance; CO_2_, carbon dioxide; CON, control group; CONSORT, Consolidated Standards of Reporting Trials; CRP, C-reactive protein; CVD, cardiovascular disease; DEXA, Dual-energy X-ray Absorptiometry; EDTA, ethylenediaminetetraacetic acid; ELISA, enzyme-linked immunosorbent assay; ELISpot, enzyme-linked immunospot; EQ-5D-5 L, EuroQol-5 dimensions, 5 levels; ESES, Exercise Self-efficacy Scale; FES, functional electronic stimulation; FSS, Fatigue Severity Scale; HDL-C, high-density lipoprotein cholesterol; HOMA, Homeostasis Model Assessment; iAUC, incremental area under the curve; IFN-γ, interferon gamma; IL-6, interleukin-6; INT, intervention group; LDL-C, low-density lipoprotein cholesterol; METs, metabolic equivalents; NEFA, non-esterified fatty acid; O_2_, oxygen; OGTT, oral glucose tolerance test; PA, physical activity; PAL, physical activity level; PBMCs, peripheral blood mononuclear cells; RCT, randomised controlled trial; RER, respiratory exchange ratio; RMR, resting metabolic rate; SCI, spinal cord injury; SF-36, short form-36; T2DM, type-2 diabetes mellitus; V̇O_2_ peak, peak oxygen uptake; WUSPI, Wheelchair User’s Shoulder Pain Index
